# Influence of PVAc/PVA Hydrolysis on Additive Surface Activity

**DOI:** 10.3390/polym12010205

**Published:** 2020-01-14

**Authors:** Ophélie Squillace, Rebecca Fong, Oliver Shepherd, Jasmine Hind, James Tellam, Nina-Juliane Steinke, Richard L. Thompson

**Affiliations:** 1 Department of Chemistry, Durham University, Stockton Road, Durham DH1 3LE, UK; fong.rj@pg.com (R.F.); olivershepherd96@gmail.com (O.S.); r.l.thompson@durham.ac.uk (R.L.T.); 2 STFC ISIS Facility, Rutherford Appleton Laboratories, Chilton, Didcot OX11 0QX, UK; jasmine.hind@stfc.ac.uk (J.H.); james.tellam@stfc.ac.uk (J.T.); nina-juliane.steinke@stfc.ac.uk (N.-J.S.)

**Keywords:** co-polymer films, additives, self-organisation, surface segregation

## Abstract

This aims to establish design rules for the influence of complex polymer matrices on the surface properties of small molecules. Here, we consider the dependence of the surface behaviour of some model additives on polymer matrix hydrophobicity. With stoichiometric control over hydrolysis, we generate systematic changes in matrix chemistry from non-polar, hydrophobic PVAc to its hydrolysed and hydrophilic analogue, PVA. With the changing degree of hydrolysis (DH), the behaviour of additives can be switched in terms of compatibility and surface activity. Sorbitol, a polar sugar-alcohol of inherently high surface energy, blooms to the surface of PVAc, forming patchy domains on surfaces. With the increasing DH of the polymer matrix, its surface segregation decreases to the point where sorbitol acts as a homogeneously distributed plasticiser in PVA. Conversely, and despite its low surface energy, octanoic acid (OA) surprisingly causes the increased wettability of PVAc. We attribute these observations to the high compatibility of OA with PVAc and its ability to reorient upon exposure to water, presenting a hydrophilic COOH-rich surface. The surfactant sodium dodecyl sulfate (SDS) does not show such a clear dependence on the matrix and formed wetting layers over a wide range of DH. Interestingly, SDS appears to be most compatible with PVAc at intermediate DH, which is consistent with the amphiphilic nature of both species under these conditions. Thus, we show that the prediction of the segregation is not simple and depends on multiple factors including hydrophobicity, compatibility, blockiness, surface energy, and the mobility of the components.

## 1. Introduction

The migration of small molecules to—and across the surface of—polymer matrices is of great significance for industries, with a wide range of applications, from food and personal care product packaging to the performance of adhesives and anti-corrosion coatings. Successful formulation rests on the ability of the additive–polymer interactions to confer the desired properties and functionality to the end products. The partitioning, segregation, and release of those small molecules in polymers govern the processing, performance, and lifetime of many industrials products [1] and are affected by factors such as temperature [2], aging, additive nature [3,4], matrix deformation [5,6], and crystallinity [7]. The process of migration, its mechanisms, molecular interactions, and timescales [8] are still not very well understood except for highly simplified model systems. Better understanding of these processes could enable prediction of product functionality, the faster innovation of new products and the avoidance of costly over-engineering and wasted resources.

**Figure 1:**
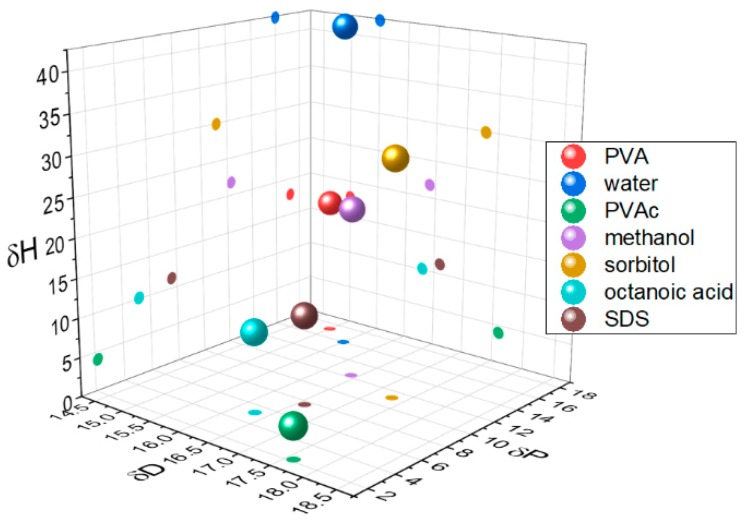
Hansen solubility parameters for polymers, additives, and solvents.

In weakly interacting systems, the ingress and swelling of glassy polymers by diffusants is well characterised by Case II diffusion [9]. Here, we are particularly concerned with the reverse case of blooming and the self-organisation of small molecules in a polymer matrix as a function of surface energy and compatibility. Compatibility can be described in terms of Hansen solubility parameters (HSP) [10], which differentiate chemicals according to their ability to interact via dispersion (δD), polar (δP), and hydrogen bonding forces (δH). In this study, we are particularly concerned with the impact of degree of hydrolysis, DH, on a poly(vinyl acetate) (PVAc) matrix, and the extent to which this can cause a mismatch between the matrix and additive properties. Increasing DH simultaneously increases the polar and hydrogen-bonding components of the Hansen solubility parameter, decreasing the hydrophobicity of the polymer (Figure 1). 

In thin films, surface and interfacial energy terms are important to the distribution of components, as these contribute significantly to the overall free energy. At a smaller degree of incompatibility than in the bulk, favourable interfaces can also promote the separation and migration of components to surfaces and interfaces if the surface energy contribution to the total free energy of the system is relatively large [11,12,13]. The fraction of migrants on the surface is determined by this equilibrium. So far, simple theories have been employed to compute the migrant fraction and layer thickness of the surface excess based on the competition between the different energies [14]. Experimental data for complex, yet well-defined systems are needed to define the influence of molecular structure on those models. For example, molecular weight (Mw) is included in basic models for compatibility such as Flory Huggins theory, but modern theories need to consider the dependence of chain flexibility, free volume, and specific interactions on mixing in order to have a useful predictive capacity [15].

Here, we consider the behaviour of migrants in matrices in which hydrophobicity is systematically varied. Hydrophobicity is well known to influence the self-organisation of the surfactants and biological molecules in processes such as micellisation, solubilisation, and protein folding, but it is normally confined to studies in aqueous solution. However, there is an interesting parallel situation where the solvent is replaced with a polymer matrix and in which the hydrophobicity can be systematically tuned. The polymers chosen for the study are relevant to many industrial formulations. Poly(vinyl acetate), “PVAc”, is a synthetic non-polar polymer and is also the precursor for its hydrolysis product, polyvinyl alcohol, “PVA”. Both polymers share excellent film-forming properties and some inherent biodegradability [16], but they have very different hydrophilicity. Hereafter, we use the notation “PVAc *X*DH”, where *X* is the percentage degree of hydrolysis. The DH is controllable and affords the possibility of a systematic variation in properties such as water-resistance, viscosity, dispersing power, tensile and adhesive strength, flexibility, and hydrophobicity [17]. Most commercial materials are either pure PVAc (*X* = 0) or commercial PVA, which is usually *X* > 85, and a variety of grades can be obtained by the controlled hydrolysis of PVAc [18]. In this work, sorbitol (S) and octanoic acid (OA) were chosen as model additives, which are widely used in industrial formulations and are known to act as plasticisers in pure PVA and PVAc, respectively [19,20]. These molecules have a similar order of molecular weight but very different hydrophilicity. As well as hydrophobic and hydrophilic molecules, we consider the influence of matrix hydrophobicity on the behaviour of an amphiphile, the anionic surfactant sodium dodecyl sulfate (SDS), which is surface active in solutions and PVA films [21]. By exploring these parameters, we show that hydrophobic interactions play an important role in the blooming of additives that are not necessarily considered to be surface active. We also make the link between the compatibility and the depth distribution of additive enriched layers, which will help the segregation behaviour of additives to be predicted and controlled in the future.

Systematic characterisation of the matrix was carried out in order to parameterise the changes occurring with the degree of hydrolysis in terms of glass transition, blockiness, and crystallinity (via differential scanning calorimetry “DSC” and ^13^C-Nuclear Magnetic Resonance, “NMR”). Plasticiser surface segregation has been quantified by nuclear reaction analysis (NRA) and neutron reflectivity (NR) for the different representative systems in the form of thin films. Thin films spin-cast from the least compatible mixtures that exhibited very irregular interfaces were not suitable for depth composition analysis, therefore: PVAc with sorbitol; PVAc (*X* > 20) with OA; and PVAc (*X* < 40) with SDS were excluded from this analysis. Although segregation is also possible at the polymer/substrate interface [22], our focus on surface exposed to air, which is common to all films, regardless of their substrate. Atomic force microscopy (AFM) enabled the characterisation of lateral phase separation and contact angles (CA) to enable quantification of the hydrophobicity of the systems. Thermal analysis was also used to understand the molecular interaction between components of the system in order to relate with the structure of the mixture and add to the full picture obtained from the lateral and depth profile data. Surface tensiometry of the water-soluble additives and PVA of high DH were measured to underpin the expected self-assembly behaviour of the systems.

## 2. Materials and Methods

### 2.1. Materials

Methanol (HPLC grade), acetone (99.5%), sulfuric acid (≥95%), HCl, (37%), potassium permanganate (KMnO4), sorbitol (S), and octanoic acid (OA) were purchased from Sigma-Aldrich, Dorset, UK. Deuterated OA (C8D15HO) was purchased from CK Isotopes (Leicestershire, UK). Deuterated sorbitol (d8-Sorbitol, C6D8H6O6) and d25-SDS were synthesised in the chemistry facility of ISIS pulsed neutron and Muon Source (Didcot, UK). PVAc of different grades of hydrolysis were prepared via the controlled hydrolysis of PVAc described by Chana et al. [18]. In our work, we found that extending the reflux time to 3 h enabled somewhat better agreement with the target value of DH than that which was originally reported. Table 1 shows a simple description of the chemical structure of the molecules: polymers and deuterated additives.

### 2.2. Sample Preparation

Stock solutions of additives and of polymers were prepared at concentrations of 2%–5% (*w*/*v*) in the relevant solvent to the system (pure methanol for PVAc; pure water for PVAc 90DH; 1:3 and 5:5 ratios of water: methanol for PVAc 40DH and PVAc 60DH, respectively). At high DH, aqueous solutions have to be heated to approximately 70 °C to dissolve PVAc 90DH. Mixed solutions of polymer and plasticiser were prepared in different ratios (2%–40% *w*/*w*). To obtain films of a suitable thickness for neutron reflectometry experiments, solutions of polymers at a concentration of 2% (low DH) and 4% (high DH) were used. These solutions were spin-cast onto 1 mm thick silicon wafers (for NRA and AFM) cut to approximately 10 × 10 mm or 5 mm thick Ø55 mm silicon blocks (for NR) using a Laurell Technologies spin coater that was set to spin at 1500 rpm (low DH) and 2500 rpm (high DH) for 45 s. The concentration, spin rate, and duration were chosen such that each solvent system had sufficient time to dry and each film appeared homogeneous, with a thickness between 100 and 200 nm (for NR) or up to 1500 nm (for NRA, AFM, and CA). Before coating, the silicon substrates were immersed in permanganic acid 2 h and rinsed thoroughly in deionised water. They were further cleaned with acetone and then placed under O_2_ plasma to remove hydrophobic impurities from the surface. Sufficient solution was pipetted onto the wafer surface to completely cover it before spinning for the wafers and for the blocks. 

### 2.3. Thermal Analysis

Following the method described by Sabattié et al. [15], samples for DSC analysis were produced by adding drops from the mixed solutions to standard DSC aluminum pans, drying each drop in an oven set to 40 °C and repeating the process until 4–6 mg of solid material had been deposited into the aluminum pan. DSC analysis of the resulting samples was run on a Perkin Elmer DSC8500 instrument using nitrogen as the purge gas at a flow rate of 20.0 mL min^−1^. Samples were heated from −25 °C to 220 °C at a heating rate of 10 °C min^−1^ or 75 °C min^−1^ with the higher rate being used to more clearly identify the glass transitions when needed. In order to ensure that the results were consistent, two heating and cooling cycles were monitored. DSC data were examined using the Pyris software, which enabled the determination of *Tg* values. Here, the *Tg* is defined as the temperature at which the peak maximum in the first derivative of heat flow with respect to temperature occurs. Estimated uncertainties are attributed to the values within a range of ±1 °C. The values are measured after the first heating and cooling cycle. Samples were prepared in the same way for thermogravimetric analysis (TGA) using a TGA Pyris 1 from 25 °C to 500 °C at a heating rate of 10 °C min^−1^ under N_2_ atmosphere.

### 2.4. Surface Tensiometry

The surface tension of aqueous solutions was measured using a KRÜSS K10 tensiometer equipped with a Du Noüy ring. The platinum ring was flame-cleaned between samples. Solutions were prepared using ultrapure water, and measurements were performed at room temperature. Repeat measurements were taken to ensure the reproducibility and accuracy of the measurements, taking into account the sensitivity of the tensiometer as well as the standard deviation defined as 0.6 mN/m. 

### 2.5. Atomic Force Microscopy

AFM was used to profile the surface topography of spin-cast films containing 10%–40% (*w*/*w*) sorbitol within host matrices of PVAc, PVAc (20, 40 DH) after spin-coating and after annealing. Annealing was achieved by heating the films to 70 °C overnight in an oven. 40 × 40 μm, 10 × 10 μm, 5 × 5 μm, and 1 × 1 μm AFM images were acquired using a Bruker^®^ Multimode 8 with Digital Instruments^®^ Nanoscope V scanning probe microscope set to operate via the Peakforce QNM in air analysis mode. ARROW-NCR-50 probes with a nominal force constant of 42 N/m were used to collect 512 × 512 pixel images. Images were analysed using Gwyddion software. This programme corrected the natural curvature of height that is caused by rastering of the film sample with respect to the cantilever via a second-order ‘flattening’. Furthermore, the software enabled in depth cross-section and roughness analysis on the scanned images. The root mean square of roughness value *R_q_* represents the standard deviation of the distribution of heights, allowing the surface roughness to be described by a statistical method and is calculated as follows:

(1) $$R_{q}=\sqrt{\overset{N}{\underset{k=0}{\sum }}\frac{1}{N}{\left(z_{k}-\bar{z}\right)}^{2}}$$

### 2.6. Contact Angle Analysis

Water contact angle measurements are the technique used to assess the affinity of the surfaces with water. Analysis was restricted to films of PVAc *X*DH, where *X* ≤ 40, since water dissolves PVA at higher DH. The surface hydrophilicity and wettability of the films were evaluated from a static contact angle by the sessile drop method using a goniometer equipped with a CCD camera and a MATLAB script for image analysis. Contact angles were measured with a precision of 0.1° each and at least 4 droplets of 1 µL were deposited on separated regions over the surface to build an average value for each sample. The contact angles were recorded immediately after droplet deposition on spin-coated films from mixed solutions. When the contact between liquid and solid is made, the measurements of the contact angle between the width of the drop and the tangent at the drop boundary were determined on both sides and averaged. Measurements were carried out for each sample at ambient air. The contact angle (θ), the diameter of the droplet area in contact with the solid (W), and its volume (V) were acquired. The variation of contact angle, width, and volume were monitored over 30 s to calculate three kinetics parameters: the velocity of angle variation ($\frac{\partial \theta }{\partial t}$), the absorption variation of the droplet (ΔV), and the spreading variation of the droplet (ΔW). The velocity of angle variation as a function of time was obtained following this equation:

(2) $$\frac{\partial \theta }{\partial t}=\frac{\theta_{\mathrm{tf}}-\;\theta_{t0}}{t_{f}-t_{0}}$$

where θ_tf_ is the contact angle at the final time t_f_ and θ_t0_ is the contact angle at the initial time t_0_. Adsorption and spreading rates were calculated using the following equations:

(3) $$\Delta V=\frac{V_{\mathrm{tf}}-{\;V}_{t0}}{V_{t0}}\;\times \;100$$

and

(4) $$\Delta W=\frac{W_{\mathrm{tf}}-{\;W}_{t0}}{W_{t0}}\;\times \;100$$

where V_tf_ and W_tf_ are the volume and width of droplet at the final time t_f_ and V_t0_ and W_t0_ are the volume and width of the droplet at the initial time t_0_.

### 2.7. Neutron Reflectometry

Specular reflectivity *R*(*Q*) was measured on the OffSpec reflectometer at ISIS pulsed neutron and muon source, Didcot, UK, from before the critical edge (*Q* ~ 0.01 Å^−1^) to the point at which background is reached (*Q* ~ 0.25 Å^−1^). Here, *Q*, the scattering vector is defined in the usual way as Q=(4π/λ)sinθ, where *λ* is the neutron wavelength and *θ* is the scattering angle. Measurement required at least two angles of incidence and typically 2 h of acquisition time per sample. The scattering length densities (SLD) of the organic components in the film are shown in Table 1. The SLD values for the silicon (2.07 × 10^−6^ Å^−2^ and the native oxide layer 3.45 ± 0.2 × 10^−6^ Å^−2^) were consistent with results that have been inferred from previous experiments on silicon substrates [3,23,24,25]. Fitting of the NR data was performed with the analysis software IGOR Pro, using the Motofit package [26] (Tables S10, S12 and S13).

The following sections, results, and discussion are based on and refer to the experiments and physicochemical characteristics that were described in the Materials and Methods section above and a Supplementary Information section where some additional values and details of the methodology are provided.

## 3. Results

### 3.1. Properties of Polymer Matrices

**Figure 2:**
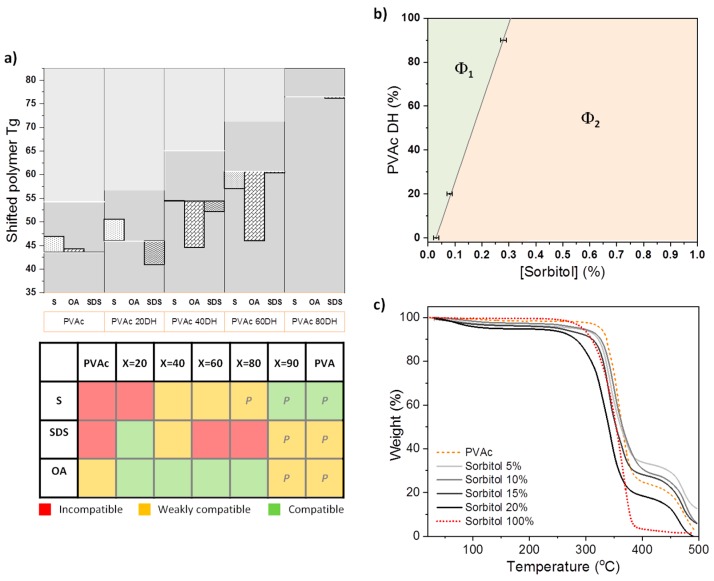
(**a**) *Tg* shifts of polymers for mixtures of sorbitol (S), octanoic acid (OA), or sodium dodecyl sulfate (SDS) with PVAc (*X* = 0, 20, 90). Horizontal white lines represent the *Tg* of pure polymer from which the *Tg* is shifted to lower (compatibility/plasticisation) or higher values (anti-plasticisation). A qualitative interpretation of the compatibility is presented in the table on the right based on the DSC results and Hansen solubility parameters (HSP) predictions. A letter P is attributed when color attributions are based only on HSP predictions. (**b**) Phase diagram of dried mixtures of sorbitol and PVAc (*X* = 0, 20, 90) obtained from cloud point analysis at room temperature, where Φ_1_ represents the one-phase region and Φ_2_ the clouded region. The technique is described elsewhere [21]. (**c**) Thermogravimetric analysis (TGA) of pure PVAc 0DH (orange dashed line) and with this addition of 5%, 10%, 15%, and 20% of sorbitol (shades of grey lines) and pure sorbitol (red dashed line).

Thermal analysis (DSC, TGA) provides quantitative and qualitative information and valuable insights into the temperature dependence of structures and interactions in materials. DSC was used to study pure PVAc *X*DH, pure additives, and their mixtures. The thermograms were monitored and the obtained glass transitions are reported in Tables S1–S6 of the Supplementary Information section (SI). The thermograms for PVAc 40DH and PVAc 90DH exhibit a relatively broad but clear melting point which indicates that sufficient intermolecular and intramolecular hydrogen bonding between hydroxyl groups exists in order for crystallite domains to form [18]. No such melting transition was observed for PVAc 0DH or PVAc 20DH, indicating that these polymers are rather amorphous. The thermograms also provide evidence of the increase of *Tg* values with the DH (Figure 2). *Tg* evolution has been shown to be correlated to the radius of cavities in the polymer matrices [27]. The decrease of *Tg*s with the increasing DH and crystallinity can suggest that the matrices at a low DH possess larger cavities than matrices at a higher DH whose radius of cavities decrease as the DH increases. Furthermore, NMR characterisation of the grades resulted in the determination of important polymeric properties such as the block character, *η*, which is defined as

(5) $$\eta =\frac{\left[\mathrm{OH}-\mathrm{Ac}\right]}{2\left[\mathrm{OH}\right]\left[\mathrm{Ac}\right]}$$

where [OH − Ac], [OH], and [Ac] are the fractional concentrations of methylene carbons between OH and acetate groups, pairs of OH groups, or pairs of acetate groups, respectively. A value of *η* < 1 corresponds to a blocky monomer distribution, which is reported elsewhere for hydrolysis products of PVAc [28]. The results show that the blockiness slightly increases with increased DH except for the commercial sample PVAc 90DH (Table 2, Figure S1 of SI for NMR methods and data). Polymers with rather low *η* values will have the majority of their hydroxyl groups associated with inaccessible crystalline regions and a small number of randomly placed, exposed vinyl alcohol units.

### 3.2. Properties of Polymer–Migrant Mixtures: Changing DH

DSC was also performed on pure sorbitol and its mixtures with polymers. For an incompatible system, the polymer *Tg* should not change once mixed with the migrant, as the polymer chains remain segregated from the smaller molecules, leaving the local environment of the amorphous regions of the polymer, and hence the free volume, unchanged. On the other hand, a change in *Tg* is expected for a compatible system, as mixing of the components leads to an adjustment in the free volume available to the polymer chains within amorphous domains [29]. The reduction of the polymer’s *Tg* is widely recognised as an indicator of plasticisation [30,31]. For PVAc at low DH, adding sorbitol slightly increases the *Tg* of the polymer (Tables S1 and S2 of the SI). We relate this increase of the polymer’s *Tg* to a state of relative compatibility, which is also referred as anti-plasticising, where both components are amorphous, making the coexistence of mixed domains possible. However, at higher DHs, the *Tg* associated with the polymer is decreased by 3.6 °C. Figure 2a illustrates the shift of the polymer’s *Tg* at different DH values in the example of 10% *w*/*w* of additive loading. It shows the increase of compatibility with sorbitol as the polymer is more hydrolysed and gets closer to the hydrophilicity of the additive. This is underpinned in Figure 2b with the phase behaviour of sorbitol in PVAc matrices at *X* = 0, 20, and 90, where the size of the single-phase region steadily increases with the DH. 

With SDS addition, Figure 2a indicates a significant change in *Tg* only for the PVAc 20DH and 40DH matrices, which suggests that the two components are compatible in this range of hydrolysis. This is consistent with the predictions from HSP (Figure 1 and Table S7 of the SI), where closer components in HSP space are more compatible. The glass transition corresponding to the PVA 87-90DH mixed system could not be detected at the applied scan rate, because this transition is masked by the evaporation of water that is typically absorbed by PVA. In the case of OA, a significant change in *Tg* is observed for the PVAc 40DH and 60DH matrices. This differs slightly from HSP predictions that predict greater compatibility with the 2 DH and 40DH.

### 3.3. Properties of Polymer–Migrant Mixtures: Changing Migrant Concentration

The influence of sorbitol concentration on the thermal stability of PVAc as a function of sorbitol loading is shown in Figure 2c. TGA reveals that the thermal decomposition of PVAc occurs in two stages. The majority of mass loss occurs at an onset of ~336 °C due to the deterioration of the acetate group leaving a percentage of residues of 30%, which is close to what was previously observed in the literature, where deterioration was shown to form C=C bonds [32]. This is followed by the decomposition of the polymer backbone at higher temperatures (452 °C) [33]. For pure sorbitol, the onset of complete deterioration occurs at 320 °C. For PVAc and sorbitol mixed samples, the thermal decomposition follows the pattern of PVAc and occurs in two stages, with a majority of weight loss occurring from ~320 °C (temperature of deterioration of pure sorbitol) to ~335 °C (temperature of deterioration of the acetate groups for pure PVAc) as the mass fraction of added sorbitol increases 5% < x < 15%. Increasing the mass fraction of sorbitol in PVAc for 0% < x < 15% decreases the thermal stability of the blend, but it is still more stable compared to the behaviour of pure PVAc, particularly for the polymer backbone. This corroborates the increase of stability suggested by DSC with the shift of *Tg*s to higher values as the concentration of additive is increased (Table S1 of the SI). Interestingly, for higher sorbitol mass fractions (>20%), the main weight loss occurs at an even lower temperature than for pure sorbitol, and the transition due to the backbone deterioration occurs at lower temperature than for pure PVAc. The mass loss occurring near 100 °C is due to water evaporation [34] and increases with the concentration of sorbitol. 

### 3.4. Surface Properties of the Films Probed through Surface Tension, CA, and AFM Measurements

**Figure 3:**
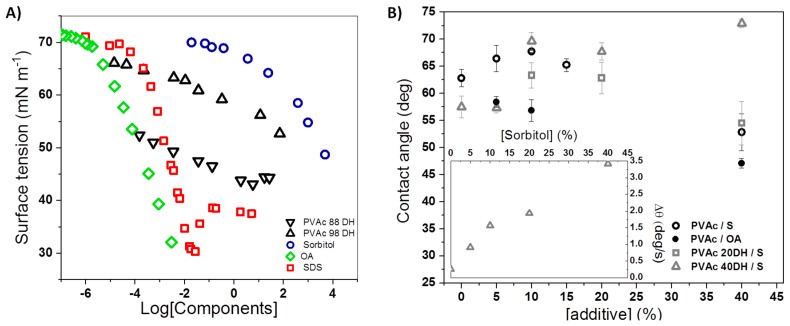
(**A**) Surface tension of PVAc 98DH, PVAc 90 DH, sorbitol, and SDS aqueous solution as a function of their concentration and of OA in 0.005 M hydrochloric acid measured by Lunkenheimer et al. [40]. (**B**) Contact angle values of PVAc, PVAc 20DH, and PVAc 40DH films as a function of sorbitol and OA mass fraction. The rate of contact angle variations over 30 s is shown in the inset as a function of sorbitol mass fraction in films of PVAc 40DH.

Surface tensiometry gives a good measure of the surface activity of solutes, which in turn guides the likely order of surface segregation during spin-casting from solution. Figure 3 shows the concentration-dependent surface tension of the three additives as well as PVA at two high DH values. In increasing order of surface activity, S < PVA 98DH < PVA 90DH < SDS < OA. The surface activity of the resin decreases with increasing DH values. Different values of the surface tension of pure PVAc are reported depending on their molecular weight and can range from 36.5 [35] to 43.3 mN m^−1^ [36]. Therefore, the surface tension of the PVAc 90DH used for this study was calculated and found to be equal to 42.8 mN m^−1^ (Table S14 of the SI), which is consistent with the limiting surface tension suggested by solutions at high concentration.

Contact angle (CA) is a common parameter to measure the degree of hydrophobicity of a material [37]. Here, we have assessed water affinity with sorbitol-plasticised PVAc films of three grades of DH: 0%, 20%, and 40%. Pure polymer surfaces with low DH are slightly hydrophilic with a contact angle that decreases when the DH is higher (θ_PVAc_ = 65°; θ_PVAc20DH_ = 57°). Upon the addition of sorbitol, the behaviour for PVAc at high DH is different to the behaviour of PVAc at low DH (Figure 3). PVAc 40 DH exhibits an increase of contact angle with increasing sorbitol concentration. In the case of PVAc, although an increase in hydrophobicity is noticed for a small fraction of sorbitol (x < 15%), once a sufficient amount of sorbitol is included, the hydrophilicity increases with the fraction of sorbitol, which was certainly due to the formation of larger sorbitol-enriched domains of amorphous PVAc and its exclusion out of the matrix to interfaces. For PVAc 20DH, a similar behaviour to the one of PVAc takes place. Due to adsorption, spreading, and evaporation [38], the CA equilibrium is not always applicable depending on the nature of the films. In this case, calculated parameters linked to the kinetics of CA can provide useful insight on the systems. Therefore, the CA, width, and volume of droplets were recorded and analysed over the first 30 s, when evaporation is negligible. The increase of droplet width in contact with the film is related to the area variation and indicates the spreading phenomenon. However, a decrease in volume corresponds to the absorption phenomenon. Although both phenomena occurred, the variation of width was greater than the decrease in volume. Therefore, the velocity of contact angle variation seen is mainly due to spreading rather than absorption [39]. The strongest variations were observed for PVAc 40DH-based films. PVAc and PVAc20 DH with a low fraction of sorbitol exhibited very negligible variations. From PVAc 40DH-based films, it is clearly seen that |−Δθ| increases with x spreading being almost linear with the mass fraction (Figure 3B, inset).

**Figure 4:**
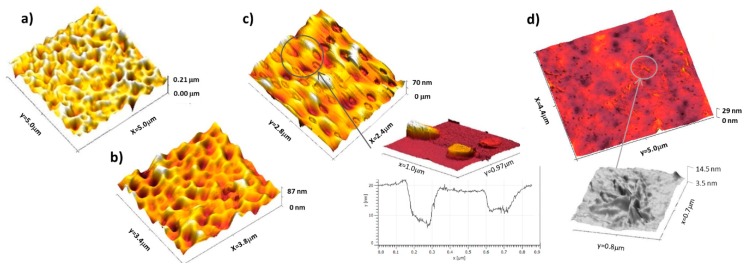
AFM height map of PVAc 0 DH films plasticised with (**a**) 5% sorbitol, (**b**) 15% sorbitol, (**c**) 40% sorbitol, and its zoomed in adhesion plot, and (**d**) 40% sorbitol after annealing overnight at 60 °C with a zoom on the ‘root-like’ surface features.

AFM measurements allow the surface topographical changes to be probed. A control experiment for pure PVAc reveals a smooth and rather featureless surface with a roughness ~2 nm for a 5 µm × 5 µm scan. PVAc films containing a low sorbitol mass fraction <10% *w*/*w* (Figure 4a) are rougher with a patchy “foam-like” structure at the interface, which can be linked to phase separation occurring along with the evaporation of the solvent [24]. Similar instabilities are observed for sorbitol-plasticised PVAc film at 20 DH. As the concentration is increased, as shown in Figure 4b,c, the porosity and grain of the surface are smaller and less defined. This is consistent with the presence of more sorbitol at the surface, as seen by CA. PVAc films plasticised with 40% of sorbitol (Figure 4d) display patchy domains of micrometric size and of few nanometers thick (3–5 nm) and root-like features were also observed after annealing [41]. 

### 3.5. Depth Profile of the Films Probed by NRA and NR

**Figure 5:**
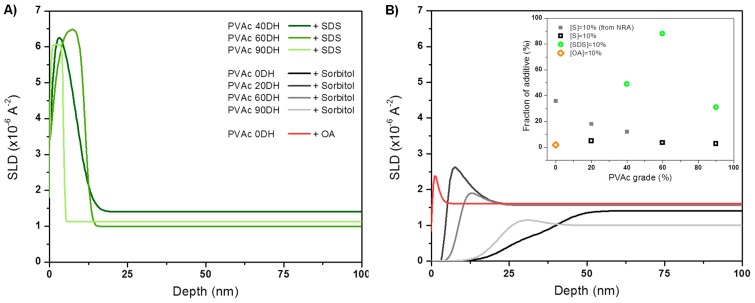
SLD profiles from NR data for (**A**) 15% d_8_-sorbitol, d_25_-SDS and (**B**) d_15_-OA loaded in PVAc matrices of different grade.

The surface segregation of plasticisers is evidenced in the films of polymer and can be quantified by the surface excess, which is defined as follows:

(6) $$z^{*}=\int_{0}^{\infty }\varphi \left(z\right)-\varphi_{b}\;dz$$

where *φ_b_* is the depth-independent bulk concentration adjacent to the surface excess region and *φ*(*z*) is the depth (*z*)-dependent volume fraction profile of the near-surface region. Fitted NR parameters are summarized in Tables S10–S13 of the SI. The surface excess is well defined by the area of the peak concentration profile, and the value of z* defines the equivalent thickness of a pure adsorbing layer. Data from NR performed on 15% (deuterated) d8-sorbitol plasticised PVAc films of different DH and fits used to obtain the SLD and concentration-depth profiles evidence a little enrichment of sorbitol at surface that decreases as the DH increases (Figure 5). None led to the formation of a surface excess bigger than the equivalent of a monolayer with values of z* equal to 0.95 nm and 0.18 nm for PVAc 60DH and PVAc 90DH, respectively (Table S11 of the SI). The thickness of the films and bulk concentration disparity could be taken into account, making the films measured from NR even more comparable, by normalising z* into a parameter *f* standing for the fraction of migrant at the surface that is plotted in the inset of Figure 5. The values confirm that no wetting layer was formed and that surface enrichment decreases as the DH increases, as it is also seen by the NRA for low DH. Indeed, NRA was used as a complementary alternative technique to NR for understanding the structure of spin-coated films having rough or irregular interfaces, and being therefore unsuitable for NR, notably PVAc/sorbitol as evidenced by AFM (Figure 4). The data and fits used to obtain the concentration-depth profiles evidence a sorbitol-enriched surface (Figure S3 of the SI) with their corresponding z* equal to 30.7 nm, 24.6 nm, and 16.7 nm for PVAc, PVAc 20DH, and PVAc 40DH, respectively (Table S8 of the SI). The estimated values of *f* for films probed by NRA at low DH (Figure S2, Table S9) are added to the plot of fractions probed by NR in the inset of Figure 5, showing the overall decrease of surface enrichment with the DH. The small discrepancy between z* values obtained from NRA and NR is related to the ratio of film thickness between films dedicated to NRA and to NR (~11) and the difference between the objects that those two techniques can probe. Films spin-coated from PVAc mixed with d_8_-sorbitol show the biggest surface enrichment. However, the very broad interface that is revealed by NRA could indicate sorbitol in deep interstices of a rough polymer surface or the co-existence of scarce sorbitol-enriched blobs amongst depleted ones. In the light of the data obtained from complementary techniques and of the work done by Negi et al. [42], it is interpreted as a grain microstructure that is somewhat enriched in sorbitol and segregated at the surface. This corroborates with the structure seen by AFM and that is suggested by the TGA analysis, as discussed further on. In the case of PVAc 0DH films mixed with a non-polar lipid (OA), NR data show that surface enrichment is lower compared to PVAc/sorbitol systems with z*(OA) = 0.34 nm (Figure 5). In the case of films mixed with a polar surfactant (SDS), there is no clear trend relating the DH of PVAc to the extent of d_25_-SDS segregation NR data show that the polymer that is almost fully hydrolysed has the lowest extent of segregation with z* = 3.2 nm, 9.6 nm, and 7.3 nm for PVAc 90DH, PVAc 60 DH and PVAc 40 DH, respectively. Over the range of DH, for which NR data could be obtained, it is clear that this additive exhibits much stronger surface segregation than sorbitol and the values of *f* along with SLD profiles suggest that this system forms wetting layers (Figure 5).

## 4. Discussion

### 4.1. Effect of Compatibility

The compatibility between the components of a binary mixture was investigated by changing the DH of PVAc and adding sorbitol. In terms of polar or hydrophobic interactions, compatibility is expected to increase gradually with increasing DH. According to the HSP analysis, hydrolysis give rise to a significant decrease of the space distances from 27.4 MPa^1/2^ (sorbitol–PVAc 0DH) to 14.3 MPa^1/2^ (sorbitol–PVAc 100DH). This calculation is consistent with our experiments: (i) The plasticising ability of sorbitol is increased with the DH (Figure 2). (ii) Evolution of the phase behaviour shows a bigger one-phase region, and therefore better compatibility, with the increase of the DH (Figure 2). (iii) The surface segregation of sorbitol decreases as the matrix is more hydrolysed (Figure 5). (iv) Unexpectedly, the surface of the binary mixture evolves from rather hydrophilic to more hydrophobic upon increasing the DH. This seems to identify the hydrophobicity mismatch or incompatibility as a driver for the presence of sorbitol on the film surface, despite having a surface tension higher than PVAc *X*DH (Figure 3). This situation can readily be explained from spreading the parameter arguments if phase separation occurs on the timescale of spin-coating and surface energy of sorbitol, $\gamma_{s}$

(7) $$\gamma_{s}<\gamma_{PVAc}+\gamma_{s-PVAc}$$

where $\gamma_{PVAc}$ is the surface energy of the polymer and $\gamma_{s-PVAc}$ is the interfacial energy between the polymer matrix and sorbitol. 

### 4.2. Insight into Molecular Interactions

We now consider each of our additive molecules in turn, in binary mixtures with PVAc *X*DH, in order to shed light on what drives the self-organisation of those different additives in PVAc-based films.

*Sorbitol.* Starting with sorbitol, the kinetics of CA and thermal analysis provide useful information when combined with the structural characteristic obtained by NRA/NR and AFM. For example, the incompatibility of the components reflects on CA variation over time. Whilst CA rapidly reaches a steady state for pure polymers and sorbitol mixed with PVAc, they vary over time when sorbitol is mixed with a higher DH of PVAc (Figure 3). This time-dependence gives an insight to the molecule present on the interface or the degree of interactions inside the bulk. For films of sorbitol mixed with PVAc 40DH, the initial high values of CA (*t*_0_) can be attributed to the low-surface energy methyl and acetyl groups that would minimise the surface component of the free energy of the film, which is initially exposed to air [13]. It is also likely that before exposure to the water droplet, intra and intermolecular hydrogen bonds between the hydrophilic –OH groups tend to minimise their exposure to the surface until some period after the water has been in contact [19]. Similar observations and an interpretation by Razavi et al. have been established for glycerol in sage seed gum films [43]. The gradual decrease of CA over 30 s is consistent with an interaction of water with hydrophilic groups inside the matrix, leading to migration or reorientation of sorbitol to the film–droplet interface (Figure 3). While sorbitol has a high surface tension against air, its solubility in water suggests that the interfacial tension with water would be very low. We note also that even if sorbitol is not initially present at the film surface, it is possible that crystalline PVA-rich domains provide compatible pathways for small molecules to migrate [7]. Hence, migration can be facilitated by the little crystallinity of the matrices at high DH, whereas low crystallinity offers free volume accessible for sorption [44]. This is consistent with our observation that the PVAc 40DH, which is more hydrophilic and blockier in nature than PVAc 0DH or PVAc 20DH, appears to facilitate the formation of a more hydrophilic interface with water. We speculate that the less blocky nature of the less hydrolysed matrices may hinder this migration, and therefore no variation of CA is seen over time. 

TGA indicates a complex behavior of partially compatible sorbitol in the homopolymer matrix, PVAc 0DH. It is apparent from Figure 2 that the interaction of sorbitol with PVAc changes the thermal property of the films. First, for mass fractions *x* <5%, the thermal stability increase of sorbitol suggests favourable molecular interactions between the components, wherein sorbitol is trapped in the matrix cavities or distributed in the polymer chains. The higher thermal stability of the polymer backbone shows that these interactions tend to favour the physical crosslinking of the matrix [45] or that sorbitol is distributed through the matrix in a way that increases inter-chain interaction [46]. Notably, fewer acetate groups are degraded, suggesting that a certain amount of them interact with sorbitol and do not degrade to lead to the formation of C=C bonds [32]. The favourable interaction of sorbitol at small loadings in the PVAc matrix, increasing the inter-chain interaction would correspond to the phenomenon known as anti-plasticisation [47]. The increase of *Tg*s observed in this system is consistent with this interpretation, since this is generally associated with a decrease in free volume [27,48,49]. 

However, when [S] in PVAc 0DH is increased to the range 5% < x < 15%, the thermal stability of sorbitol decreases, along with the stability of the polymer backbone. We speculate that sorbitol aggregates into a separate phase at these higher loadings, given that its –OH groups are more likely to interact with each other than with the PVAc groups [19,50,51,52]. Further increasing [S] will have the effect of increasing the size of these aggregates among polymer chains, possibly contributing to a porous structure which, when the sorbitol degrades, is more susceptible to thermal degradation than the pure material would have been. This corroborates the surface properties probed by CA and AFM. The surface of PVAc films becomes more hydrophilic as the mass fraction of sorbitol is increased. This suggests that the system goes from a state where sorbitol fills cavities, squeezing the polymer chains, to a state where sorbitol is more drastically phase separated from the PVAc and excluded to the interfaces (Figure 4). At high sorbitol concentration in PVAc, where a significant incompatibility is suggested by DSC and phase diagrams (Table S1 and Figure 2), nanometric features are visible by AFM (Figure 4). Incompatibility and (where applicable) crystallinity can drive molecular migration toward the surface (sorbitol >20% *w*/*w* in PVAc), suggesting that it is more energetically expensive for the small molecules to be alongside the polymer chains than in contact with air. This process required early phase separation, before total evaporation of the solvent, leaving sorbitol at the top of the surface after total evaporation. 

*Sodium dodecyl sulfate.* In the polymer matrices, SDS segregate to a much greater extent than sorbitol and form wetting layers. There are likely to be a number of influencing factors contributing to this segregation behaviour. One possible factor is the surface energy differences between the resins. With its high surface activity, SDS is expected to enrich the surface of the solution during spin-casting. From Figure 3, it is observed that there is a greater difference between the surface energy of the components with a higher DH of PVAc. On this basis, a higher degree of segregation would be expected with a resin of a higher degree of hydrolysis (PVAc 90DH). This is contrary to what is observed (Figure 5), indicating that while surface tension has a role in directing surface segregation, it is not a good indicator of the extent of segregation. 

The complex variation in surface excess values as a function of matrix DH indicates that multiple factors are simultaneously involved in the development of this surface. Our DSC analysis indicates through the magnitude of *T*_g_ shift that most of the DH PVAc values are quite incompatible with SDS, except for PVAc 20DH. This is similar to what HSP reveal, where a clear minimum is seen in the HSP space, nearer X = 40DH than X = 20DH with accounts for uncertainty and a lack of blockiness consideration. The apparent peak in z*(SDS) for PVAc 60DH cannot be ascribed to this alone, since one would expect that while it is less compatible with SDS than PVAc 20DH or PVAc 40DH, it does not then explain the lower z* value found for PVAc 90DH. We note that the PVAc 90DH is not in the same series as PVAc 0–60DH, and as such has a different and lower extent of blockiness as well as a lower molecular weight. We can speculate that one of these features is the origin of the anomalously low z*; e.g., that being less blocky, there are more hydroxyl groups free to interact favourably with the SDS headgroups [21,53], or that it has a greater solubility, so it does not exclude the SDS until a later stage of spin-coating the film. Previously, we have established that the presence of an additional plasticiser can lead to a significant increase in z*(SDS) in PVA 90DH; therefore, it is likely that z*(SDS) in these unplasticised films may be limited by the kinetics of segregation as much as thermodynamics of miscibility [21]. 

*Octanoic Acid.* To explore whether surface segregation is driven primarily by incompatibility or surface tension, we finally consider the behavior of OA in PVAc. Similar to SDS, OA has an amphiphilic structure. OA has a very low surface tension, which was reported at 27.5 mN m^−1^ for the pure component [54] and even greater surface activity in aqueous solutions than SDS (Figure 3). Interestingly, no large surface excess of OA in PVAc 0DH was measured (Figure 5), which is reminiscent of the most compatible mixtures of sorbitol with PVAc 90DH. This indicates that in the absence of some incompatibility, surface segregation is greatly reduced. Interestingly, OA still modifies the surface property of the film in a surprising way. Contact angles are lower with added OA than with added sorbitol (Figure 4), which is counter-intuitive, since segregation the low surface energy additives are normally associated with highly hydrophobic surfaces. This surface wetting is attributed to the rapid switching of OA orientation. Presumably, OA adsorbs to the PVAc surface spontaneously by lowering the surface energy when the octyl chains are outermost. However, in contact with water, OA could reorient so that the hydrophobic octyl group faces the hydrophobic PVAc surface, and the hydrophilic –COOH group is presented to the interface with water: a surface-chemical ‘win–win’. 

## 5. Conclusions

Tuning of the surface properties of PVAc films via the DH is an easy to control parameter with significant consequences for surface properties and molecular organisation. However, the prediction of the segregation is not simple and depends on multiple factors including the hydrophobicity, compatibility, blockiness, surface energy, and mobility of components. We have shown that additive concentration can also induce changes in the structure, surface properties, and even thermal stability. 

PVAc films mixed with additives such as sorbitol, OA, and SDS were used to explore the effect of the degree of hydrolysis on surface segregation in order to provide further insight into the role of compatibility and polymer–surfactant interactions on segregation. In all cases, incompatibility seems to be critical for surface segregation, ahead of surface tension or molecular weight, but a significant modification of surface properties can be achieved at low levels of surface activity. Looking at the effect of DH gave insight into the origins of incompatibility. This compatibility can be related to hydrophobicity, matching molecules with their host matrix, as well as more subtle factors such as blockiness and amphiphilicity. While in the case of a polar oliol such as sorbitol, compatibility is easily related to the DH and therefore hydrophobicity mismatch; however, it is more complex in the case of amphiphilic molecules such as SDS where incompatibility is intrinsically linked with the matrix structure and blockiness. The study of two surface-active migrants with alkyl tails but different polar headgroups has shown that OA is accommodated better with PVAc than SDS. Finally, thermal analysis, AFM, CA, and IBA have proved to be powerful complementary tools for giving insight into polymer–additive interactions and structure, enabling better understanding of the structure property relationships that are necessary for computational approaches to predict the behavior of additives in complex polymer matrices.

## Figures and Tables

**Table 1:** Physicochemical characteristics of the components such as molecular weight (Mw), scattering length density (SLD), and chemical structure. Molecules with an asterisk designate commercial components. SLDs of the synthesised polymer were calculated via a statistical average of the SLDs for pure poly(vinyl acetate) (PVAc) and polyvinyl alcohol (PVA).

Component	Chemical Structure	M_W_/g mol^−1^	SLD/×10^6^ Å^−2^
PVAc 0DH *	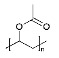 or 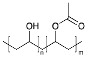	100,000 - - - 30,000–70,000 89,000–98,000	1.31
PVAc 20DH PVAc 40DH			1.23
			1.14
PVAc 60DH			1.03
PVAc 90DH * PVAc 98DH *			0.78
			-
d8-Sorbitol	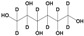	190.2	4.985
d-15 OA	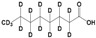	159.3	6.1
d25-SDS	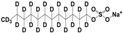	313.3	6.77

**Table 2:** Degree of hydrolysis (DH) for each grade of PVAc and its corresponding glass transition measured by differential scanning calorimetry (DSC) and blockiness obtained by ^13^C NMR.

Predicted DH	Actual DH	*Tg*/C	*η*
0	0	44 ± 1	-
20	20.0 ± 0.8	46 ± 1	0.44 ± 0.05
40	39 ± 1	51 ± 1	0.35 ± 0.05
60	59.4 ± 0.7	60.7 ± 0.5	0.33 ± 0.01
80	79 ± 1	76.5 ± 0.5	0.32 ± 0.02
88	85.7 ± 0.6	50 ± 2	0.49 ± 0.02

## Supplementary Materials

The following are available online at https://www.mdpi.com/2073-4360/12/1/205/s1, Table S1: Glass transitions for mixed PVAc and sorbitol samples at 10 °C/min, Table S2: Glass transitions for mixed PVAc 20 DH and sorbitol samples at 10 °C/min, Table S3: Glass transitions for mixed PVAc 40 DH and sorbitol samples at 75 °C/min, Table S4: Glass transitions for mixed PVAc 60DH and sorbitol samples at 10 °C/min, Table S5: Glass transitions for mixed PVAc XDH and OA (x = 10% *w*/*w*) samples and their shift compared to pure polymer at 10 °C/min, Table S6: Glass transitions for mixed PVAc XDH and SDS (x = 10% *w*/*w*) samples and their shift compared to pure polymer at 10 °C/min, Table S7: Summary of distances between components in Hansen solubility parameter space obtained with the software HSPiP, Table S8: Equivalent thickness of a pure adsorbing layer *z** and its corresponding fractions of segregated additive *f* calculated from data obtained by NRA on PVAc films of different grades mixed with 10% of sorbitol, Table S9: Best fits parameters for the NRA data measured at a take-off angle of 70° on films of PVAc, PVAc 20DH, and PVAc 40DH mixed with 15% of d8-sorbitol, Table S10: SLD of the different grades of PVAc and silicium as the substrate on which the binary films were spun for NR experiments, Table S11: Equivalent thickness of a pure adsorbing layer *z** and its corresponding fractions of segregated additive *f* calculated from data obtained by NR on PVAc films of different grades mixed with 10% sorbitol, or 10% OA, or 10% SDS, Table S12: Best-fit parameters for the NR data measured on films of PVAc, PVAc 20, 60, and 90DH mixed with 15% of d8-sorbitol, Table S13: Best fits parameters for the NR data measured on films of PVAc mixed with 15% of d_15_-OA; and PVAc 20, 60, 90DH mixed with 15% of d_25_-SDS, Table S14: Contact angles and different surface energy components to the contribution of the surface energy of the solid film’s surface of PVAc, Figure S1: One of the ^13^C NMR spectra obtained for PVAc 40DH. The inset shows the methylene region of the spectrum, along with the chemical environments of each methylene carbon, Figure S2: NRA data, fits, and composition profiles for 15% d_8_-sorbitol loaded in PVAc, PVAc 20DH, and PVAc 40DH thin films, Figure S3: NR data and fits from thin films of (A) 15% d_8_-sorbitol; (B) d_25_-SDS and d_15_-OA; added to PVAc of different grades.
