# Supplementary material for: Influence of PVAc/PVA Hydrolysis on Additive Surface Activity
Source: Polymers (Basel). 2020 Jan 14;12(1):205. doi: 10.3390/polym12010205 (PMC7023474; doi:10.3390/polym12010205)
Supplement: Supplementary file 1 [file polymers-12-00205-s001.pdf]

Supplementary Materials

Influence of PVAc / PVA hydrolysis on Additive Surface Activity.

Ophélie Squillace*[1], Rebecca Fong[1], Oliver Shepherd[1], Jasmine Hind[2], James Tellam[2], Nina-Juliane Steinke[2] and Richard L. Thompson[1].

[1]Department of chemistry, Durham University, Stockton road, Durham, UK.
[2]STFC ISIS Facility, Rutherford Appleton Laboratories, Chilton, Didcot, OX11 0QX, U.K.

Table of Contents:

1. Thermal analysis 2
2. Hansen Solubility Parameters (HSP) 3
3. Nuclear Magnetic Resonance 4
4. Depth profile analysis 5
   4.1 Nuclear Reaction Analysis 5
   4.2 Neutron Reflectometry 6
5. Surface energy calculation for PVAc 7

1. Thermal analysis

DSC measures the temperatures and heat flows associated with transitions in materials as a function of temperature or time in a controlled atmosphere. This technique provides quantitative and qualitative information about physical and chemical changes that involve endothermic or exothermic processes or changes in heat capacity as the material is heated. DSC was used to assess the compatibilitiy of pure compounds and mixtures (PVAc of different grades, sorbitol and their mixtures). The thermograms were monitored and their glass transition reported in Tables 1 to 5. Estimated uncertainties of ±1°C are attributed to the values.

Table S1 : Glass transitions for mixed PVAc and Sorbitol samples at 10°C/min

Weight fraction of sorbitol (%)	$T_{g1,x}$ (PVAc) (°C)	$T_{g2,x}$ (Sorbitol) (°C)
0	43.6	
10	46.9	5.1
20	48.2	5.0
40	49.1	5.2
100		1.2

Table S2 : Glass transitions for mixed PVAc 20DH and Sorbitol samples at 10°C/min

Weight fraction of sorbitol (%)	$T_{g1,x}$ (PVAc 20DH) (°C)	$T_{g2,x}$ (Sorbitol) (°C)
0	46.0	
10	50.6	32.0
20	51.4	21.8
40	47.3	12.1
100		1.2

Table S3 : Glass transitions for mixed PVAc 40DH and Sorbitol samples at 75°C/min

Weight fraction of sorbitol (%)	$T_{g1,x}$ (PVAc-40DH) (°C)	$T_{g2,x}$ (Sorbitol) (°C)
0	54.4	
10	54.3	54.3
40	52.7	22.7
100		7.1

Table S4 : Glass transitions for mixed PVAc 60DH and Sorbitol samples at 10°C/min

Weight fraction of sorbitol (%)	$T_{g1,x}$ (PVAc-60DH) (°C)	$T_{g2,x}$ (Sorbitol) (°C)
0	60.7	
10	47.1	47.1

Table S5 : Glass transitions for mixed PVAc XDH and OA (x=10%w/w) samples and their shift compared to pure polymer at 10°C/min

PVAc grade (%)	$T_{g1,x}$ (°C)	ΔT_g (°C)
0	43.6	-0.7
40	48.2	9.8
60	49.1	14.7

Table S6 : Glass transitions for mixed PVAc XDH and SDS (x=10%w/w) samples and their shift compared to pure polymer at 10°C/min

PVAc grade (%)	$T_{g1,x}$ (°C)	ΔT_g (°C)
0	44	0
20	41	5
40	49.1	1.9
60	60.4	0.3
80	76.2	0.3

2. Hansen Solubility Parameters (HSP)

Table S7. Summary of distances between components in Hansen Solubility Parameter space obtained with the software HSPiP ($\sqrt{4\delta D^2 + \delta P^2 + \delta H^2}$)

species	PVAc	PVAc 20DH	PVAc 40DH	PVAc 60DH	PVAc 80DH	PVAc 90DH	PVA
sorbitol	27.4	23.9	20.7	17.8	15.6	14.8	14.3
SDS	9.3	5.2	1.9	3.9	7.9	10.0	12.0
OA	7.3	3.7	3.1	6.4	10.4	12.4	14.5

3. Nulear Magnetic Resonance

^{13}C NMR Sample Characterisation. PVAc samples were prepared for NMR analysis by dissolving 50 mg of each grade in 0.8 ml of the relevant deuterated mixed solvent combination at 80 °C with stirring. Deuterated solvents were required in order to avoid shimming and so that the NMR machine (Bruker (UK)) could stabilise the magnetic field strength. The resultant solutions were placed into clean NMR sample tubes (5 mm) and subsequently analysed by the Durham University Solution State NMR service. The ^{13}C NMR experiments were proton decoupled, conducted at a frequency of 100 MHz and lasted for approximately 55 minutes. Three repeat spectra were recorded for each grade such that the standard error corresponding to the DH and block character of each sample could be determined. The subsequent NMR spectra were analysed using MestReNova v12.0.0 software. The precise DH of each synthesized product along with the supplied sample of PVA 87-90DH was determined via ^{13}C NMR. Samples were prepared for NMR analysis as described in section 3.3. Figure 4.1shows a typical full ^{13}C NMR spectrum obtained for PVAc 40DH accompanied by an enlarged view of the methylene region, which contained the three peaks that were required to calculate the value of DH. In each sample, these peaks occurred at approximately 45 ppm (OH-OH dyad), 42 ppm (OH-Ac dyad) and 38 ppm (Ac-Ac dyad). For perspective, the chemical environments of these dyads in the context of a PVAc chain are shown in Figure S-2. For all spectra, the relative intensities of the three peaks were calculated as percentages of the combined peak heights using the MestReNova software integration tool. These relative intensities were then used to calculate the DH via the following equation[1]: DH = (OH-OH + [OH-Ac]/2)x100. Table 3 of the main document summarises the ^{13}C NMR data collected for each hydrolysed sample along with the uncertainty in the determined value of DH. This uncertainty represents the standard error in the relative intensities of the three dyads and the resultant DH as three repeat measurements were conducted for each PVA/c grade in order to ensure that the hydrolysis synthesis was reproducible and consistent across each of the samples. The standard error was calculated via the following equation $\sigma_x = \sigma/n^{1/2}$; where σ_x is the standard error, σ the standard deviation of the sample values and η the number of samples. The block character of the polymer was calculated along with this equation: η = [OH-Ac]/(2[OH][Ac]).

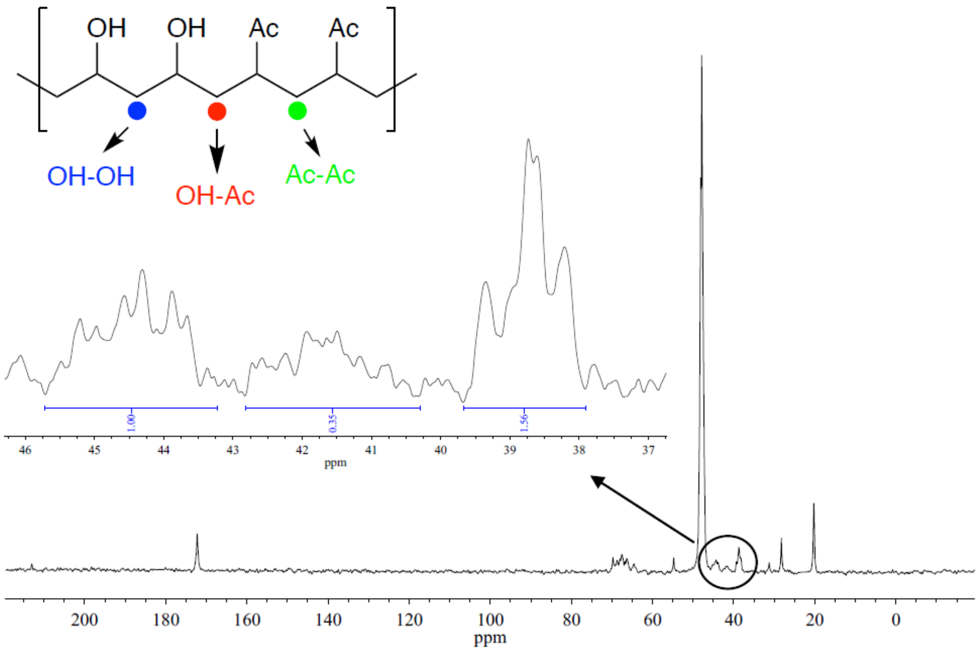

Figure S1 : One of the 13C NMR spectra obtained for PVAc 40DH. The inset shows the methylene region of the spectrum, along with the chemical environments of each methylene carbon.

4. Depth profile analysis

4.1 Neuclear Reaction Analysis

The application of IBA techniques including nuclear reaction analysis (NRA) to soft matter have been described previously and can also be found in greater detail elsewhere[2-3]. As IBA operates under vacuum conditions (<4×10^{-6} Torr), loss of low-molecular-weight components from polymer films can occur; hence, liquid-nitrogen sample cooling to below -50°C[4] was required to vitrify the samples before analysis under vacuum. NRA relies on contrast to identify the depth distribution of components in films via deuterium labelling. Deuterium labelled OA and Sorbitol were therefore used to isolate the depth distribution of individual components. When performing NRA under described experimental conditions a resolution of 8 nm is achieved. Although insufficient to give orientational features it is sufficient to quantify surface segregation of the deuterated plasticizer and quantification of surface enrichment is possible even if films are too rough to address with NR. IBA data were analysed with the Surrey University DataFurnace software (WiNDF v9.3.68 running NDF v9.6a) to determine the concentration versus depth profile, where the densities of PVAc XDH and d8-OA were assumed to be ~ 1.19 g cm^{-3} and ~ 0.91 g cm^{-3}; respectively. In order to avoid over-parametrisation, model composition profiles were restricted to a few layers, for which the composition and thickness was allowed to vary to obtain the best possible fit to the experimental data. The fitting procedures used for DataFurnace are described in detail elsewhere[5].

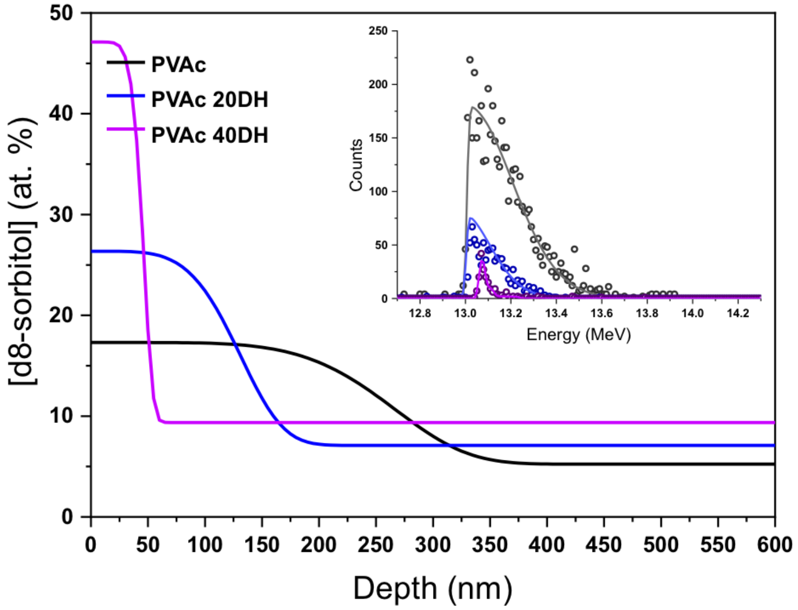

Figure S2. NRA data, fits, and composition profiles for 15% d$_8$-sorbitol loaded in PVAc, PVAc 20DH, and PVAc 40DH thin films

Table S8. Equivalent thickness of a pure adsorbing layer z* and its corresponding fractions of segregated additive *f* calculated from data obtained by NRA on PVAc films of different grades mixed with 10% of sorbitol. [a] Due to substantial uncertainties in film thickness, fractions were estimated for films of 1100nm.

NRA	z*(nm)
PVAc XDH	+ d-8 Sorbitol
0	30.7 / 35.8[a]

20	24.6 / 18.0[a]
40	16.7 / 12.0[a]

Data from NRA were fitted using the following equation for sorbitol concentration (at. %):

$$[Sorbitol] = A_0\, e^{\left(\frac{d}{A_1}^{A_2}\right)} + A_3, \quad (1)$$

The best fit parameters obtained from the data measured at a take-off angle of 70° on three films of PVAc, PVAc 20DH and PVAc 40DH uploaded with 15% d8-sorbitol are reported in the following table:

Table S9. Best fits parameters for the NRA data measured at a take-off angle of 70° on films of PVAc 0, 20 and 40DH mixed with 15% of d8-sorbitol

NRA	A_0	A_1	A_2	A_3	χ^2
PVAc	0.6	2790	5.2	0.26	3.9
PVAc 20DH	0.4	1.389	4.5	0.14	0.49
PVAc 40DH	0.2	457.7	7.0	0.05	0.17

4.2 Neutron Reflectometry

The following figure shows the reflectivity data obtained from films of PVAc at 0, 20, 40, 60 and 90% DH mixed with 15% of d_8-sorbitol, d_{25}-SDS and d_{15}-OA. The calculated SLD expected for the different polymers: PVAc and PVAc/PVA copolymers of 20, 60 and 90DH used to initialize the fits of NR data are reported in the Table S-10. The best-fit parameters for the corresponding data are reported in Tables S-11 and S-12. Finally the equivalent pure layer z* and fraction of migrant at the surface are summarized in Table S-13. The scope of this paper only focuses on the surface enrichment at the air/polymer interface but does not exclude the formation of a wetting layer et the substrate/polymer interface. Therefore, fits were systematically restricted the same way for this latter interface to avoid compensation effects and have a good comparison of the structure at the surface exposed to air between the different systems studied.

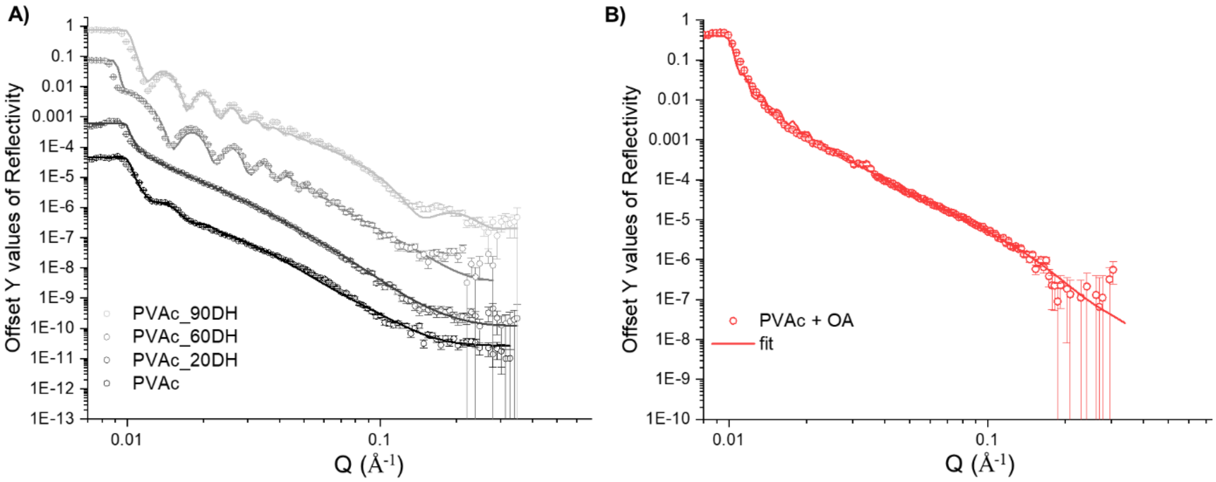

Figure S3. NR data and fits from thin films of A) 15% d_8-sorbitol; B) d_{25}-SDS and d_{15}-OA; added to PVAc of different grades

Table S10. SLD of the different grades of PVAc and silicium as the substrate on which the binary films were spun for NR experiment

NR	SLD (×10^{-6} Å^{-2})
PVAc	1.310
PVAc 20DH	1.182
PVAc 40DH	1.06
PVAc 60DH	9.30
PVAc 90DH	7.34
PVA	6.70
Si	2.07

Table S11. Equivalent thickness of a pure adsorbing layer z* and its corresponding fractions of segregated additive *f* calculated from data obtained by NR on PVAc films of different grades mixed with 10% of sorbitol, or 10% of OA, or 10% of SDS.

NR	z*(nm) / *f*(%)		
PVAc XDH	+ d_8- Sorbitol	+ d_{25}-SDS	+ d_{15}-OA
0			0.34[b] / 2.7
20	2.2 / 4.9		
40		7.3 / 49	
60	0.95 / 3.4	9.6 / 88	
90	0.18 / 2.7	3.2 / 31	

Table S12. Best fits parameters for the NR data measured on films of PVAc 0, 20, 60 and 90DH mixed with 15% of d8-sorbitol (d, the film thickness; SLD, the scattering length density; and σ, the surface roughness)

	PVAc _ d_8-S			PVAc 20DH _ d8-S			PVAc 60DH _ d8-S			PVAc 90DH _ d8-S		
Front.	d (Å)	SLD (×10^{-6} Å^{-2})	σ (Å)	d (Å)	SLD (×10^{-6} Å^{-2})	σ (Å)	d (Å)	SLD (×10^{-6} Å^{-2})	σ (Å)	d (Å)	SLD (×10^{-6} Å^{-2})	σ (Å)
1	2	2.67	159	41	3.47	13	56	2.21	21	15	2.34	27
2	878	1.38	117	4088	1.58	65	628	1.55	55	859	0.87	34
3	48	4.3	46	16	1.64	15	23	3.00	55	32	2.24	7
4	2.3	3.47	8	10	3.47	9	4	3.47	5	19	3.47	5
Back.	$\chi^2 = 30$			$\chi^2 = 65$			$\chi^2 = 541$			$\chi^2 = 214$		

Table S13. Best fits parameters for the NR data measured on films of PVAc mixed with 15% of d_{15}-OA; and PVAc 20, 60, 90DH mixed with 15% of d_{25}-SDS (d, the film thickness; SLD, the scattering length density; and σ, the surface roughness)

	PVAc _ d_{15}-OA			PVAc 40DH _ d_{25}-SDS			PVAc 60DH _ d_{25}-SDS			PVAc 90DH _ d_{25}-SDS		
Front.	d (Å)	SLD (×10^{-6} Å^{-2})	σ (Å)	d (Å)	SLD (×10^{-6} Å^{-2})	σ (Å)	d (Å)	SLD (×10^{-6} Å^{-2})	σ (Å)	d (Å)	SLD (×10^{-6} Å^{-2})	σ (Å)
1	9	3.57	6	85	6.77	15	114	6.51	27	45	6.06	4
2	1869	1.6	18	1318	1.40	37	1072	0.99	14	1040	1.12	4
3	1	4.7	19	13	3.47	7	10	3.47	10	12	3.41	9
4	6	3.47	5									
Back.	$\chi^2 = 108$			$\chi^2=20$			$\chi^2= 20$			$\chi^2= 36$		

5. Surface energy calculation for PVAc

The surface energy of PVAc was calculated using van Oss' extension and Young's theory and three following solvents: water, formamide, diiodomethane, with their corresponding thermodynamic parameters[6]. The average of contact angles values obtained from the different solvents on PVAc film and the calculated surface tensions are reported in the following table.

Table S14. Contact angles and different surface energy components to the contribution of the surface energy of the solid film's surface of PVAc (γ_{SV}^{LW} the apolar contribution to the surface energy of the solid; γ_{SV}^{-} and γ_{SV}^{+} the electron-donor and electron-acceptor contributions to the polar surface energy component of the solid; γ_{SV} the surface energy of the solid)

CA			Surface tension components (mJ/m^2)			
Water	Formamide	Diiodomethane	γ_{SV}^{LW}	γ_{SV}^{-}	γ_{SV}^{+}	γ_{SV}
66.4	50.4	39.0	39.7	15.2	0.16	42.8

References

(1) Chana, J.; Forbes, B.; Jones, S. A., The synthesis of high molecular weight partially hydrolysed poly (vinyl alcohol) grades suitable for nanoparticle fabrication. *Journal of nanoscience and nanotechnology* **2008,** *8* (11), 5739-5747.

(2) Jeynes, C.; Colaux, J. L. J. A., Thin film depth profiling by ion beam analysis. **2016,** *141* (21), 5944-5985.

(3) Composto, R. J.; Walters, R. M.; Genzer, J. J. M. S.; Reports, E. R., Application of ion scattering techniques to characterize polymer surfaces and interfaces. **2002,** *38* (3-4), 107-180.

(4) Clough, A.; Collins, S.; Gauntlett, F.; Hodgson, M.; Jeynes, C.; Rihawy, M.; Todd, A.; Thompson, R. J. J. o. m. s., In situ water permeation measurement using an external 3He2+ ion beam. **2006,** *285* (1-2), 137-143.

(5) Barradas, N.; Jeynes, C. J. N. I.; Materials, M. i. P. R. S. B. B. I. w.; Atoms, Advanced physics and algorithms in the IBA DataFurnace. **2008,** *266* (8), 1875-1879.

(6) van Oss, C. J. J. J. o. M. R., Long-range and short-range mechanisms of hydrophobic attraction and hydrophilic repulsion in specific and aspecific interactions. **2003,** *16* (4), 177-190.
